# Endocrine Therapy for Endometrial Carcinoma: Current Evidence, Resistance Mechanisms, and Biomarker-Driven Patient Selection

**DOI:** 10.3390/curroncol33020124

**Published:** 2026-02-19

**Authors:** Taro Yamanaka, Hiroshi Yoshida, Tatsunori Shimoi, Kazuki Sudo, Kan Yonemori

**Affiliations:** 1 Department of Medical Oncology, National Cancer Center Hospital, Tokyo 104-0045, Japan; tayaman2@ncc.go.jp (T.Y.); tshimoi@ncc.go.jp (T.S.); ksudo@ncc.go.jp (K.S.); kyonemor@ncc.go.jp (K.Y.); 2 Department of Diagnostic Pathology, National Cancer Center Hospital, Tokyo 104-0045, Japan

**Keywords:** endometrial cancer, estrogen receptor, progesterone receptor, endocrine therapy, progestin, selective estrogen receptor degraders, CDK4/6 inhibitor, mTOR inhibitor

## Abstract

The treatment landscape for endometrial carcinoma (EC) is undergoing a paradigm shift from traditional histopathological dualism to precision medicine grounded in the Cancer Genome Atlas (TCGA) molecular classification. The “No Specific Molecular Profile” (NSMP) subgroup, the largest molecular cohort, has emerged as a particularly promising target for endocrine-based strategies. While endocrine therapy (ET) has been a mainstay for over 60 years due to its favorable safety profile, its efficacy as monotherapy remains modest. This review provides a comprehensive overview of current endocrine strategies, including traditional agents like progestins and aromatase inhibitors, and focuses on novel combination therapies designed to overcome resistance. Recent clinical trials have demonstrated that integrating molecularly targeted agents, such as CDK4/6 and mTOR inhibitors, significantly improves clinical outcomes. Specifically, patients with *TP53* wild-type status and *CTNNB1* mutations exhibit exceptional responses to these combinations. Furthermore, we discuss the potential of next-generation selective estrogen receptor degraders (SERDs) and the importance of refining patient selection through robust predictive biomarkers. Driven by molecular insights, endocrine therapy is transitioning from a secondary palliative option into a definitive cornerstone of precision oncology, offering a personalized and effective treatment for patients with advanced or recurrent endometrial carcinoma.

## Simple Summary

Endometrial cancer is the most common gynecological cancer in developed countries, with a rising global incidence. Traditionally, endocrine therapy served as a low-toxicity alternative to chemotherapy, although its efficacy as monotherapy was often limited. Recently, however, promising results have been reported for combinations involving endocrine therapies and molecularly targeted agents. Additionally, advances in molecular classification suggest the existence of specific patient populations highly responsive to endocrine therapies. This review synthesizes current evidence regarding endocrine monotherapy and combination regimens, exploring resistance mechanisms and optimal patient selection. By evaluating ongoing clinical trials and future prospects, this review serves as an important educational resource for clinicians seeking to understand endocrine therapy-based treatment strategies, including emerging combination approaches, within the evolving landscape of endometrial cancer treatment.

## 1. Introduction

Endometrial carcinoma (EC) is a malignant tumor originating from the endometrial epithelium. It is the most common gynecological cancer in developed countries, with its incidence and mortality rates increasing annually [1]. The increasing incidence of EC is associated with multiple factors, including an aging population, obesity, type 2 diabetes, early menarche, and delayed menopause [2,3]. More than 80% of endometrial cancers are estrogen receptor positive and associated with estrogen related risk factors such as obesity, nulliparity, late menopause, early menarche, and menopausal estrogen supplementation [4]. Notably, the incidence and mortality rates of young-onset EC continue to rise in some countries including Japan and the United States [5]. While patients with early-stage EC who are eligible for curative resection have a favorable prognosis [6], recurrent disease remains difficult to eradicate, with a poor 5-year survival rate of 20–25% [7]. Although recent years have seen the emergence of new therapeutic strategies for advanced or recurrent EC, including immune checkpoint inhibitors combined with chemotherapy [8,9,10] and novel molecularly targeted agents [10,11,12], the improvement in overall prognosis remains insufficient. Furthermore, low-grade tumors are known to be less sensitive to cytotoxic chemotherapy [13], necessitating the development of alternative treatment strategies.

For many years, EC was classified into Types I (estrogen-dependent) and II (estrogen-independent) proposed by Bokhman in 1983 [14]; however, the ProMisE classification is now widely utilized, and molecular classification is recommended for all EC cases [15]. Immunohistochemical (IHC) assessment of ER expression is also recommended [15], as it serves as a prognostic factor within the “no specific molecular profile” (NSMP) group and as a predictive biomarker for the efficacy of endocrine therapy in the advanced or recurrent setting [15]. Simultaneously, the evaluation of the progesterone receptor (PR) is advocated [16].

Compared to cytotoxic chemotherapy, endocrine therapy offers a lower toxicity profile and may be a viable option for patients deemed unfit for chemotherapy. The sensitivity of EC to hormonal manipulation has been established for over 60 years, dating back to the 1960s [17]. Current guidelines continue to recognize endocrine therapy as a valid treatment option for patients with low-grade, hormone receptor-positive, or asymptomatic, slow-growing advanced/recurrent EC [15]. While progestins, aromatase inhibitors, selective estrogen receptor modulators (SERMs), and selective estrogen receptor degraders (SERDs) have been utilized, recent clinical trials have focused on combination therapies with molecularly targeted agents.

In this review, we summarize the current knowledge regarding endocrine therapy for EC. Our discussion encompasses traditional agents such as progestins, aromatase inhibitors, and SERMs, as well as novel SERDs and combinations with molecularly targeted drugs, including their molecular biological rationales. Furthermore, we explore the potential application of endocrine strategies proven effective in other malignancies, such as breast cancer, to the management of EC. Finally, we offer perspectives on the future role of endocrine therapy in the evolving treatment landscape of endometrial carcinoma. Notably, this review does not address hormonal therapies used for fertility-sparing purposes.

## 2. Current Knowledge Around Endocrine Therapies

### 2.1. Pathological Diagnosis

The classification system for endometrial carcinoma has undergone significant evolution from a purely histopathological framework to a molecularly defined taxonomy, aiming to refine prognostic stratification and guide more precise therapeutic decision-making [15,18,19,20]. For many years, endometrial carcinoma was classified according to the dualistic model proposed by Bokhman in 1983, which stratified tumors primarily based on histologic features and clinical behavior [14]. In this model, Type I (estrogen-dependent) tumors account for approximately 80–85% of cases, are typically low-grade (grade 1/2) endometrioid carcinomas, are associated with unopposed estrogen exposure and obesity, and have generally been considered to carry a favorable prognosis [14,21]. By contrast, Type II (estrogen-independent) tumors comprise about 15–20% of cases and include grade 3 endometrioid carcinomas and non-endometrioid histotypes such as serous carcinoma, clear cell carcinoma, and carcinosarcoma, which are generally associated with an adverse prognosis [14,21]. However, this dichotomous model has important limitations: it shows suboptimal reproducibility and does not capture the considerable prognostic heterogeneity, particularly within high-grade tumors [22,23]. In 2013, the Cancer Genome Atlas (TCGA) Research Network published a comprehensive genomic characterization of endometrial carcinoma using next-generation sequencing and integrative analysis of somatic mutations and copy-number alterations [18]. This work led to the identification of four prognostically relevant molecular subgroups, marking a paradigm shift from traditional histotype-based classification to a molecular classification scheme [18]. Analyzing cohorts of endometrioid and serous carcinomas, TCGA defined the following four molecular subtypes: POLE-ultramutated (POLEmut): tumors with an ultrahigh mutational burden (>100 mutations/Mb), associated with an excellent prognosis. Microsatellite instability-high (MSI-H)/hypermutated (mismatch repair–deficient, MMRd): tumors with a high mutational burden (approximately 10–100 mutations/Mb), associated with an intermediate prognosis. Copy number-low (CN-low): tumors with a low mutational burden and few copy-number alterations; because they lack defining molecular markers, they are referred to as NSMP and are associated with an intermediate prognosis. Copy number-high (CN-high)/p53-abnormal (p53abn): tumors characterized by extensive copy-number alterations and frequent TP53 mutations, associated with the poorest prognosis [18].

**Figure 1:**
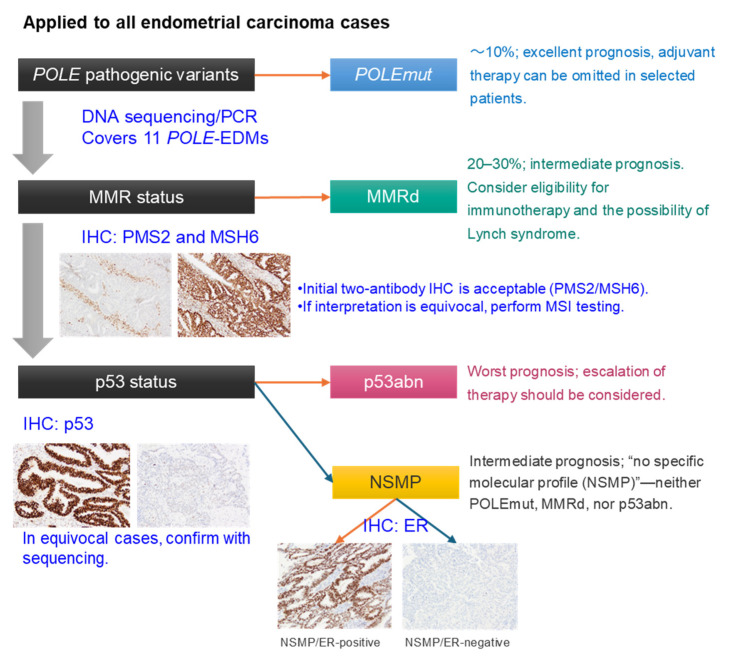
Practical workflow of the TCGA-surrogate molecular classification approach. The molecular classification of endometrial carcinoma is illustrated according to the 2025 ESGO/ESTRO/ESP guidelines. All endometrial carcinomas, regardless of histologic subtype, are candidates for molecular classification. The first step is to assess *POLE* exonuclease domain mutations (currently, at least 11 pathogenic variants have been described) to identify the *POLE*mut subgroup. The next step is immunohistochemistry for mismatch repair (MMR) proteins to identify the MMRd subgroup, followed by p53 immunohistochemistry to categorize p53abn and NSMP tumors. Further subclassification of NSMP based on ER immunohistochemistry into ER-positive and ER-negative NSMP has also been proposed. Tumors with multiple classifiers are assigned according to the highest-priority upstream classifier—for example, a tumor harboring both a *POLE* mutation and MMR deficiency is classified as *POLE*mut.

Following the landmark TCGA study, efforts were made to translate NGS-based molecular classification into routine clinical practice by developing and validating more rapid, cost-effective surrogate marker approaches (Figure 1) [24,25,26,27]. The ProMisE classifier integrates immunohistochemical (IHC) assessment of mismatch repair (MMR) proteins (MSH6, PMS2, MLH1, and MSH2) and p53, together with targeted sequencing of POLE exonuclease domain hotspot mutations, to identify the four molecular subtypes [24,25,26,27]. The prognostic performance of this methodology has been validated across several large cohort studies and clinical trials (e.g., the PORTEC cohorts), demonstrating high concordance with the TCGA classification [19,25,26,28]. Furthermore, in the translational research analysis of the PORTEC-3 trial [19], this molecular framework demonstrated exceptionally strong prognostic value in high-risk endometrial carcinoma. Specifically, the p53abn subtype was associated with poor outcomes (5-year recurrence-free survival: 48%), whereas the POLEmut subtype exhibited an extremely favorable prognosis (5-year recurrence-free survival: 98%) [19]. In contrast, the largest group, NSMP, demonstrated heterogeneous clinical behavior, prompting efforts for further risk stratification. Grade and ER expression were subsequently identified as key prognostic determinants within NSMP, with Grade 1–2 and ER-positive NSMP showing a 5-year disease-specific mortality of only 1.6%, similar to POLEmut [29]. Based on this accumulating evidence, the 5th edition of the WHO Classification (2020) formally incorporated the molecular classification (POLEmut, MMRd, p53abn, NSMP) into the taxonomic framework of endometrial carcinoma, establishing it as a standard component of pathological diagnosis worldwide [30]. Building upon this foundation, further evidence supporting the clinical utility of molecular classification continued to accumulate. In the revised FIGO staging system introduced in 2023, molecular classification, together with anatomical factors, histologic subtype, and lymphovascular space invasion (LVSI), was integrated to refine prognostic stratification [20]. Notably, in early-stage endometrial carcinoma (Stages I and II), molecular classification can directly alter stage assignment. For example, tumors of the POLEmut subtype confined to the uterus are down-staged to Stage IAmPOLEmut regardless of LVSI status or histologic subtype, reflecting their excellent prognosis [20]. Conversely, tumors of the p53abn subtype with myometrial invasion are up-staged to Stage IICmp53abn irrespective of cervical stromal invasion or LVSI, in recognition of their poor prognosis [20]. Although molecular classification does not change the anatomical stage in Stage III and IV disease, its documentation is recommended to inform prognostic evaluation and therapeutic decision-making (e.g., Stage IIImMMRd, Stage IVmp53abn) [20]. Despite the formal incorporation of molecular classification into an international staging system, implementation remains challenging in resource-limited settings, and some organizations, such as the ICCR, currently take a cautious stance [31], deferring mandatory application of molecular classification due to feasibility and complexity concerns.

However, molecular classification is now being actively leveraged as a predictive biomarker to guide therapeutic decision-making in both adjuvant treatment and systemic therapy for advanced or recurrent endometrial carcinoma [15,32]. One representative example is treatment individualization in the adjuvant setting. Molecular analysis of the PORTEC-3 trial demonstrated that the p53abn subgroup derives a significant survival benefit from the addition of chemoradiotherapy (CRT), the POLEmut subgroup achieves excellent outcomes with radiotherapy (RT) alone, and the MMRd subgroup gains no benefit from CRT [19]. These findings established the biological rationale for tailoring adjuvant therapy based on molecular subtype. Building on this foundation, ongoing de-escalation trials, such as PORTEC-4a (NCT03469674), are testing molecular profile-guided treatment assignments in patients with intermediate- and high-risk endometrial carcinoma [33]. Furthermore, it is now well established that MMRd status is a strong predictive biomarker for response to immune checkpoint inhibitors (ICIs), reshaping frontline systemic therapy for advanced or recurrent endometrial carcinoma [8,9,10,15,32,34].

In parallel with the mounting clinical trial evidence and advances in molecular pathology, major international clinical guidelines have positioned molecular classification as a core determinant of therapeutic decision-making [15,32]. The ESGO/ESTRO/ESP guidelines, published in 2021 and subsequently updated in 2025 [15], recommend universal molecular classification for all endometrial carcinomas and incorporate molecular subtype into a risk-stratification algorithm [15]. The 2025 update further recommends ER immunohistochemistry for prognostic stratification within the NSMP subgroup [15]. Likewise, the latest NCCN guidelines (Version 3.2025) strongly recommend molecular assessment using POLE mutation testing, MMR/MSI evaluation, and p53 immunohistochemistry to complement morphologic diagnosis [35]. Taken together, the classification of endometrial carcinoma has undergone a paradigm shift, from the historically broad Type I/II framework to a precision oncology-oriented system grounded in TCGA genomic data and enabled in routine clinical practice by surrogate markers [7,15,18,19,20,25]. This evolution now allows refined prognostication and evidence-based therapeutic selection across the disease continuum.

#### 2.1.1. ER Signaling and the Proportion of ER Positive Cases

**Figure 2:**
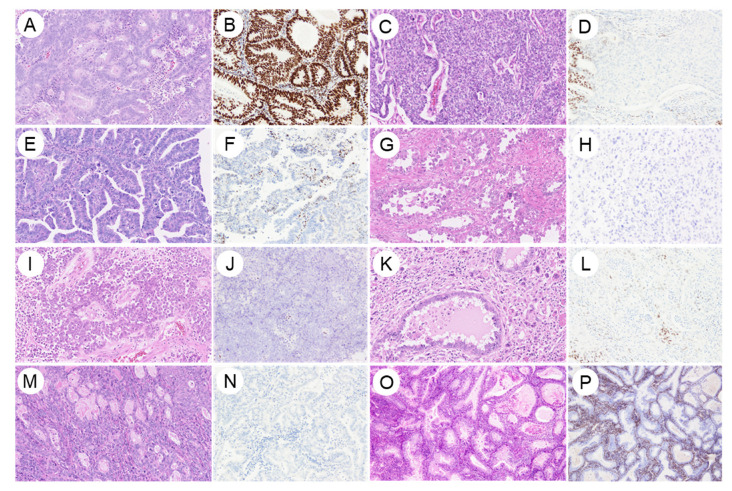
Estrogen receptor expression across histological subtypes of endometrial cancer. Representative examples of ER expression in major histologic subtypes of endometrial carcinoma are shown. (**A**,**B**) Endometrioid carcinoma (Grade 1); (**C**,**D**) Endometrioid carcinoma (Grade 3); (**E**,**F**) Serous carcinoma; (**G**,**H**) Clear cell carcinoma; (**I**,**J**) Dedifferentiated carcinoma; (**K**,**L**) Carcinosarcoma; (**M**,**N**) Mesonephric-like adenocarcinoma; (**O**,**P**) Gastric-type adenocarcinoma. (**A**–**P**), ×200.

The biological role of ER signaling in endometrial carcinoma is fundamentally linked to tumorigenesis and cancer proliferation [36,37]. ER signaling has long been recognized as the cornerstone of EC pathogenesis within the traditional Type I (estrogen-dependent) classification [14,38]. Estrogen acts through ER to promote proliferation of the endometrial epithelium, exerting a mitogenic effect. In the setting of estrogen excess, such as in obesity, where peripheral aromatization of androgens to estrogens in adipose tissue leads to chronically elevated estrogen exposure, unopposed estrogen signaling stimulates ER-mediated cellular proliferation and is thought to drive carcinogenesis [39,40]. Most tumors within NSMP, the largest molecular subgroup of EC, are hormonally driven and indolent, consistent with the prototype of Type I carcinomas [29,41,42]. At the molecular level, ER promotes cancer growth through hormone-dependent transcriptional regulation, representing a biologically tractable therapeutic target [42]. The proportion of ER-positive cases in each histological type and molecular classification subtype (Table 1) and representative histological examples for each histological type are shown in Figure 2.

#### 2.1.2. Hormonal Receptor Expression as Prognostic Biomarker

Although ER expression has historically been regarded as clinically relevant in earlier diagnostic and classification systems, it has now emerged as a decisive prognostic determinant within the molecular era, particularly for risk stratification of the NSMP subtype [58,59,60]. The NSMP group accounts for approximately 40–50% of EC diagnoses and represents a molecularly heterogeneous population with intermediate outcomes [29,60]. ER expression has been incorporated into contemporary international guidelines as a robust prognostic variable for stratifying risk within the NSMP group. Supporting evidence comes from multiple studies and meta-analyses demonstrating that ER positivity in NSMP EC is strongly associated with significantly reduced recurrence risk (hazard ratio 0.37, corresponding to a 63% reduction) and improved survival (hazard ratio 0.22). In particular, patients with Grade 1–2 and ER-positive NSMP tumors are now identified as “low-risk NSMP,” characterized by a 5-year disease-specific mortality rate of only 1.6%, lending strong support to the consideration of adjuvant treatment de-escalation strategies (e.g., omission of adjuvant therapy) [29,60]. Conversely, patients with Grade 3 and/or ER-negative NSMP tumors are categorized as “high-risk NSMP” and account for most disease-specific deaths within this subgroup [60]. ER negativity is an established, independent adverse prognostic biomarker across EC broadly, and is frequently associated with unfavorable molecular profiles (e.g., p53abn) and adverse histopathologic features [46,47,48]. Notably, the prognostic impact of ER status persists even after stratification by molecular subtype (MMRp, MMRd, p53abn) and by ESGO/ESTRO/ESP risk classes [58,59,60].

### 2.2. Endocrine Therapies for Endometrial Carcinoma (Table 2)

Endocrine therapies currently utilized in clinical practice include progestins, medroxyprogesterone acetate (MPA) and megestrol acetate (MA), aromatase inhibitors (letrozole and anastrozole), and tamoxifen [15]. According to the ESGO–ESTRO–ESP guidelines, endocrine therapy is the preferred systemic treatment for patients with low-grade, estrogen receptor-positive disease, particularly those with low-volume or asymptomatic tumors, or in cases of advanced or slowly progressing recurrent disease [15].

**Table 2:** Endocrine therapies for endometrial carcinoma.

Category	Agent (Dose/Schedule)	ORR (%)	CBR (%)	Median PFS (mo)	Median OS (mo)	Reference
Monotherapy						
Progestin	MA 800 mg/d	24%	46%	2.5	7.6	[61]
	MPA 200 mg/d	25%	N/A	3.2	11.1	[62]
	MPA 1000 mg/d	15%	N/A	2.5	7.0	[62]
	Meta-analysis (26 trials)	30%	52%	N/A	N/A	[63]
SERM	TAM 40 mg/d	10%	N/A	1.9	8.8	[64]
AI	Anastrozole 1 mg/d	9%	44%	3.2	N/A	[65]
	Letrozole 2.5 mg/d	9.4%	44%	3.9	8.8	[66]
	Exemestane 25 mg/d	10%	N/A	3.1	10.9	[67]
SERD	Fulvestrant 250 mg IM	11.4%	34.3%	2.3	13.2	[68]
	Imlunestrant	10.3%	33%	3.8	N/A	[69]
Dual Combinations						
Dual Endocrine therapy	MA 160 mg/d and tamoxifen 20 mg /d	19%	N/A	N/A	8.6	[70]
	MPA 200 mg and tamoxifen 40 mg, alternating weekly	33%	N/A	3	13	[71]
	Tamoxifen 40 mg days 1–28, and MPA 200 mg days 8–14 and 22–28, in a 28-day cycle	25%	69%	4	17	[72]
Endocrine therapy and mTOR inhibitor	Letrozole + Everolimus	22%	78%	6	31	[72]
	Anastrozole + Vistusertib	24.5%	N/A	5.2	N/A	[73]
Endocrine therapy and CDK4/6 inhibitor	Letrozole + Ribociclib	10%	N/A	5.4	15.7	[74]
	Letrozole + Abemaciclib	30%	46.7%	9.1	N/A	[75]
	Fulvestrant + Abemaciclib	44%	68%	9.0	N/A	[76]
	Letrozole + Palbociclib	9%	N/A	8.3	N/A	[77]
	Imlunestrant + Abemaciclib	18.2%	42.4%	6.8	N/A	[69]
Triple Combination						
Endocrine therapy + Targeted	Letrozole + Abemaciclib + Metformin	32%	60%	19.4	N/A	[78]

Abbreviations: ORR, objective response rate; CBR, clinical benefit rate; PFS, progression-free survival; OS, overall survival; MA, megestrol acetate; MPA, medroxyprogesterone acetate; SERM, selective estrogen receptor modulator; TAM, tamoxifen; AI, aromatase inhibitor; SERD, selective estrogen receptor degrader.

#### 2.2.1. Progestins

Progestational agents have been recognized for their clinical efficacy in EC for more than 60 years [17]. Although it has been hypothesized that progesterone exerts its therapeutic effects primarily through the progesterone receptor (PR) [79], the precise molecular mechanism of action remains elusive [70]. In the GOG 121 trial, MA at a dose of 800 mg/day was evaluated in 63 patients with advanced or recurrent EC who were ineligible for local therapy and had received no prior cytotoxic or hormonal therapy [1,61]. The overall response rate (ORR) was 24% (complete response, 11%; partial response, 13%), and 22% of patients achieved stable disease (SD), resulting in a clinical benefit rate (CBR) of 46% [61]. Notably, the ORR for patients with histological grade 1 or 2 disease (37%) was significantly higher than that for those with more poorly differentiated tumors (8%; *p* = 0.02) [61]. The median progression-free survival (PFS) and overall survival (OS) were 2.5 and 7.6 months, respectively [61]. Although the frequency of grade 3 or higher adverse events was low, weight gain and hyperglycemia were observed [61]. In the GOG 81 trial, 299 patients with advanced or recurrent EC who were ineligible for local therapy were randomized to receive MPA at a dose of either 200 mg/day or 1000 mg/day [62]. The ORR was 25% in the low-dose group and 15% in the high-dose group. The median PFS and OS were 3.2 months and 11.1 months in the low-dose arm, respectively, compared to 2.5 months and 7.0 months in the high-dose arm [62]. Although the primary objective of this study was to determine whether high-dose MPA would yield superior response rates, no such benefit was observed. Rather, the findings suggested that low-dose MPA remains a more reasonable clinical option [62].

#### 2.2.2. Tamoxifen

Tamoxifen is the most utilized selective estrogen receptor modulator (SERM) for breast cancer and has been studied in recurrent/metastatic endometrial cancer. In a Gynecologic Oncology Group study (GOG 81F) which included patients with advanced or recurrent EC, the ORR was 10% and the median PFS was 1.9 months [64]. Although patients with histological grade 1 or 2 disease exhibited higher response rates than those with grade 3 disease [64], the treatment overall demonstrated only modest clinical activity.

#### 2.2.3. Tamoxifen + Progestin

Given the modest efficacy of endocrine monotherapy and the necessity to overcome treatment resistance, the development of novel therapeutic strategies was required. Based on the understanding that progesterone acts through PR, and reports indicating that short-term administration of tamoxifen significantly increased PR levels in postmenopausal women with EC [80], clinical trials were conducted to evaluate the synergistic effect of combining progestin and tamoxifen [64]. A clinical trial by the Eastern Cooperative Oncology Group (ECOG) initially planned to compare megestrol monotherapy with a combination of megestrol and tamoxifen (megestrol 80 mg and tamoxifen 10 mg twice daily) [70]. However, the monotherapy arm was terminated early due to poor accrual, precluding a direct comparison between the two groups [70]. In the combination arm (n = 42), the ORR was 19% (complete response, 1 [2%]; partial response, 7 [17%]), which was comparable to historical monotherapy data, and the median survival was 8.6 months [70]. Notably, severe complications, including life-threatening pulmonary thromboembolism, were observed more frequently in the combination group than in historical monotherapy cohorts [70]. The GOG also conducted trials on combination therapy [71,81]. In a phase II study (GOG 119) of MPA and tamoxifen (MPA 200 mg and tamoxifen 40 mg, alternating weekly), 58 patients with metastatic or recurrent endometrial cancer were treated, yielding an ORR of 33%, a median PFS of 3 months, and a median OS of 13 months [71]. A post hoc analysis using specimens from enrolled patients revealed that ER expression was significantly associated with clinical response [82]. In the GOG 153 trial, which utilized MA and tamoxifen (MA 160 mg and tamoxifen 40 mg, alternating every 3 weeks), the ORR was 27%, with a median PFS of 2.7 months and a median OS of 14.0 months among 56 evaluable patients [81]. Among patients with Grade 1 disease, the ORR reached 38% [81]. Although common adverse events in both trials, such as weight loss and thromboembolism, were considered feasible [83], it remained unclear whether the addition of tamoxifen to progestins provided a definitive clinical advantage [83]. Recently, a non-comparative randomized controlled trial (GOG 3007) adopted a combination of MPA and tamoxifen (tamoxifen 40 mg days 1–28, and MPA 200 mg days 8–14 and 22–28, in a 28-day cycle) [72]. The ORR was 25%, and at a median follow-up of 37 months, the median PFS was 4 months (5 months for chemotherapy-naïve patients and 3 months for those previously treated with chemotherapy) [72]. The study concluded that this regimen demonstrated clinically meaningful efficacy [72].

#### 2.2.4. Aromatase Inhibitors

Aromatase is highly expressed within the endometrial stroma, where it facilitates the local biosynthesis of estrogen, potentially driving estrogen-mediated neoplastic proliferation [84]. Aromatase inhibitors (AIs) have been demonstrated to inhibit proliferation and induce apoptosis in endometrial cancer cell lines in vitro [66]. Given their established efficacy and superiority over tamoxifen in ER-positive breast cancer, AIs have garnered significant interest in the management of endometrial cancer [65]. In a GOG phase II trial, anastrozole was evaluated in 23 patients with advanced or recurrent endometrial carcinoma (grade 2, n = 9; grade 3, n = 14) for whom local therapy was not curative. Eligible patients had received no prior chemotherapy and no more than one prior hormonal regimen. The study reported an ORR of 9%, a median PFS of 1 month, and a median OS of 6 months, indicating only minimal clinical benefit [85]. Conversely, the PARAGON phase II trial investigated anastrozole in 84 hormone-naïve patients with ER- and/or PR-positive endometrial cancer. While the ORR was 9%, the CBR at 3 months reached 44%, with a median PFS of 3.2 months. Notably, this clinical benefit was associated with clinically significant improvements in quality of life (QOL) [65]. Letrozole, another AI, was evaluated in a Canadian phase II trial involving patients with advanced or recurrent disease who were ineligible for curative local therapy and were naïve to both chemotherapy and prior hormonal therapy (though prior progestin use was permitted). The ORR in this cohort was 9.4% [66]. Although ER and PR expression were confirmed in evaluable tumor tissues, no significant correlation was observed between receptor status and clinical response [66]. The placebo-controlled, double-blind, randomized phase II ENGOT-EN3/PALEO trial further examined letrozole in patients with recurrent endometrioid endometrial cancer that was ER-positive (≥10% expression by immunohistochemistry) and had received at least one prior systemic therapy [77]. In the placebo plus letrozole arm (n = 37), the median PFS was 3.1 months, and the ORR was 16% [77]. Finally, an open-label, one-arm, two-stage phase II study conducted by the Nordic Society of Gynecologic Oncology evaluated exemestane in 51 patients with advanced or recurrent endometrial carcinoma, including both ER-positive and ER-negative cohorts. Among ER-positive patients, the ORR was 10%, and the median PFS was 3.8 months, whereas no responses (ORR 0%) were observed in the ER-negative group [67].

#### 2.2.5. Fulvestrant

Fulvestrant is an ER antagonist without known agonistic properties that downregulates cellular levels of ER and has become an established drug in the treatment of postmenopausal women with hormone receptor-positive advanced breast cancer [86]. In a phase II trial involving 35 patients with EC (ER and/or PR positive or unknown), the ORR was 11.4%, with a median OS of 13.2 months [68]. In a clinical trial conducted by the GOG, among 31 ER-positive patients, one (3%) achieved a complete response, four (13%) had a partial response, and nine (29%) had stable disease [79]. Conversely, among 22 ER-negative patients, no objective responses were observed [79]. In a single-arm phase II study (the FUCHSia study), four patients with EC were enrolled and received fulvestrant at a dose of 250 mg every 28 days [87]. All patients had previously been treated with aromatase inhibitors or progestins and were ER-positive (defined as ≥10% immunoreactivity in tumor cells). The median PFS was 14 weeks, and no objective responses were observed, as the best overall response for all patients was progressive disease [87].

In summary, as endocrine monotherapy or dual endocrine combinations yield only modest antitumor activity, the integration of molecularly targeted agents has emerged as a promising strategy to overcome resistance and enhance therapeutic efficacy.

### 2.3. Mechanisms of Resistance and Knowledge Gaps

Mechanisms of endocrine therapy resistance are well described in hormone receptor-positive breast cancer, whereas those contributing to endocrine therapy failure in endometrial cancer remain incompletely defined. Acquired resistance may involve genomic changes in ER signaling, including *ESR1* mutations and reduced or lost ER expression, potentially enabling ligand-independent ER activity. Non-genomic escape may occur through activation of alternative pathways (e.g., *HER2*, *FGFR*, insulin-like growth factor 1) that stimulate *PI3K/AKT/mTOR* and/or *MAPK* signaling. Dysregulated cell-cycle control (cyclin D1 upregulation and increased CDK4/6 activity) and tumor microenvironmental changes have also been implicated [88].

Other resistant mechanisms include loss of ER expression, *PI3K* pathway alterations, the receptor tyrosine kinase pathway (i.e., *FGFR1, 2* and *ERBB2* alterations) and the *MAPK* pathway (*KRAS* mutation), all of which are upstream of the cyclin D1/ CDK4/6 complex [89].

#### 2.3.1. Hormonal Receptor Expression as Predictive Biomarker

A systematic review and meta-analysis of progestin therapy for advanced and recurrent EC, encompassing 26 trials and 1639 patients, has been conducted [63]. The analysis reported that the ORR of progestin therapy was 30% (95% CI 25–36), and the clinical benefit rate was 52% (95% CI 42–61). Notably, the ORR reached 55% in PR-positive EC, whereas it was only 12% in PR-negative disease (risk difference 43%, 95% CI 15–71) [63]. These findings suggest that PR expression is a potential predictive biomarker for the efficacy of progestin therapy. Furthermore, histological tumor grade was also found to be significantly associated with the clinical response to progestins [63].

#### 2.3.2. ESR1 Mutations

Across published cohorts, *ESR1* mutations appear to be infrequent in endometrioid EC. One study reported an overall frequency of 4.0% (113/2851) [90]. In that dataset, among 120 pathogenic *ESR1* variants, the distribution was dominated by Y537S (n = 36), followed by Y537N (n = 18), D538G (n = 18), and L536H (n = 4) [90]. The same report noted a higher prevalence in metastatic/recurrent disease compared with primary tumors (7.6% vs. 3.4%, *p* < 0.001) [90].

A separate large-scale analysis described an overall *ESR1* mutation rate of 2.38% (of 17,666 cases), with endometrioid histology showing greater enrichment than other subtypes (4.2% vs. 1.7%, *p* < 0.05) [91]. In that report, *ESR1*-mutant tumors were associated with lower TP53 mutation rates (10.7% vs. 50.9%) but higher frequencies of PI3K-pathway alterations, including *PTEN* (70.4% vs. 40%), *PIK3R1* (34.1% vs. 19.6%), *PIK3CA* (47.9% vs. 36.6%), and *AKT1* (11% vs. 2.8%), together with increased *CTNNB1* (52.5% vs. 13.9%) and *ARID1A* mutations (56% vs. 32.2%) [91]. *ESR1* mutations also appeared more frequently among patients with prior aromatase inhibitor exposure (6.38% vs. 2.65%, q = 0.02) [91].

Another cohort estimated the overall *ESR1* mutation prevalence at 2.0% (63/3101) [92]. Notably, *ESR1*-activating mutations frequently co-occurred with *mTOR/PIK3CA*-pathway genomic alterations; in one series, 99% of such cases carried at least one of these events (*PTEN* 75%, *PIK3CA* 56%, *PIK3R1* 42%, *AKT1* 12%) [90]. In NSMP endometrial cancer, *ESR1* mutations were reported in 3.6% (9/253) [93].

## 3. Recent Progress in Endocrine Therapy

### 3.1. Combination Strategies (Table 2)

#### 3.1.1. Combination with PI3K/AKT/mTOR Pathway Inhibition

The *PI3K/AKT/mTOR* pathway is involved in the development of EC [94]. Hyperactivation of *PI3K/AKT/mTOR* signaling has been associated with advanced disease and poor prognosis across all histological subtypes of EC [95], leading to its emergence as a significant therapeutic target [96]. However, the efficacy of mTOR inhibitor monotherapy has been limited, with ORR ranging from 0% to 14% [97]. Furthermore, as the *PI3K/AKT/mTOR* pathway has been implicated in conferring resistance to hormonal therapy [96], clinical trials evaluating combination strategies have been conducted. A phase II trial investigating the efficacy and safety of the mTOR inhibitor temsirolimus, either as monotherapy or in combination with hormonal therapy (alternating MA and tamoxifen), was terminated early due to a high incidence of venous thromboembolism in the combination arm. Moreover, the addition of hormonal therapy failed to enhance clinical activity in that study [97]. In contrast, GOG 3007, a non-comparative randomized controlled trial, evaluated letrozole plus everolimus versus hormonal therapy (tamoxifen and MPA). While the ORRs were 22% and 25%, and the median PFS was comparable at 6 and 4 months, respectively, the letrozole plus everolimus arm demonstrated a remarkably prolonged median PFS of 28 months in chemotherapy-naïve patients (compared to 4 months in pretreated patients) [72]. Based on these findings, it is highly probable that distinct mechanistic differences exist between the interactions of mTOR inhibitors with progestins versus aromatase inhibitors [97]. Additionally, the VICTORIA phase I/II trial evaluated the combination of the mTOR inhibitor vistusertib and anastrozole versus anastrozole monotherapy in 73 patients with advanced or recurrent ER+ and/or PR + EC who had received up to one prior line of chemotherapy. The median PFS was 5.2 months for the combination versus 1.9 months for monotherapy, and the ORR was 24.5% versus 17.4%, respectively, suggesting that the addition of an mTOR inhibitor provides superior clinical benefit over hormonal therapy alone [73].

#### 3.1.2. Combination with CDK4/6 Inhibitors

In endometrioid EC, aberrations in the *PI3K* pathway and the *RTK/RAS-CTNNB1* pathway are observed in approximately 90% and 80% of cases, respectively [18]. Both pathways are suggested to contribute to endocrine therapy resistance [98,99] by inducing ER-independent upregulation of cyclin D1 (CCND1), a primary ER target gene essential for estrogen-mediated cell proliferation [98,99]. The upregulation of CCND1 activates cyclin-dependent kinases 4 and 6 (CDK4/6), which in turn promotes cell cycle progression through the phosphorylation of the retinoblastoma protein (RB1). This phosphorylation leads to the dissociation of RB1 from the E2F transcription factor, thereby upregulating the transcription of genes involved in the G1-to-S phase transition [99]. These molecular findings have provided a rationale for investigating the combination of CDK4/6 inhibitors and hormonal therapy [75].

In a phase II trial evaluating the combination of letrozole and the CDK4/6 inhibitor ribociclib in 20 patients with ER-positive endometrial cancer, the progression-free rates were 55% at 12 weeks and 35% at 24 weeks [74]. More favorable outcomes were specifically observed in patients with grade 1–2 EC [74]. The study noted that the selection threshold for ER expression (≥10% immunoreactivity) was somewhat arbitrary. Consequently, it was concluded that further research is necessary to determine whether a statistically significant correlation exists between the quantitative level of ER expression and the clinical response to this combination therapy [74].

In a phase II trial evaluating the combination of letrozole and a CDK4/6 inhibitor abemaciclib in 30 patients with ER-positive recurrent endometrial cancer (of whom 28 had endometrioid histology), the regimen demonstrated favorable clinical activity [75]. The ORR was 30%, the CBR was 46.7%, and the median PFS was 9.1 months [75]. Notably, clinical efficacy was observed irrespective of PR expression or MMR status [75]. Exploratory analyses revealed a median PFS of 9.1 months in patients with *TP53* wild-type tumors, compared to only 2.3 months in those with *TP53* mutations (*p* = 0.026) [75]. Furthermore, mutations in *CTNNB1, KRAS,* and *CDKN2A* were suggested as potential predictive biomarkers of treatment response [75]. The overall safety profile was consistent with previous trials in breast cancer and was considered manageable [75]. Regarding abemaciclib, a phase II trial evaluated its combination with fulvestrant in patients with advanced or recurrent endometrial cancer. Among 25 patients with ER- or PR-positive disease (defined as ≥1% expression by immunohistochemistry), the ORR was 44%, and the median PFS was 9.0 months [76]. The safety profile was considered manageable. Notably, molecular correlation analysis revealed that 10 of the 11 responders (91%) belonged to the NSMP subgroup; within this subgroup specifically, the ORR reached 59%. In contrast, no objective responses were observed among patients with *TP53*-mutated tumors [76].

The double-blind, randomized phase II ENGOT-EN3/PALEO trial investigated the efficacy of letrozole in combination with the CDK4/6 inhibitor palbociclib in patients with ER-positive endometrioid EC [77]. The study demonstrated a statistically significant prolongation of median PFS in the combination arm, which reached 8.3 months compared to 3.1 months in the letrozole monotherapy arm (HR 0.56). Regarding safety, a higher incidence of Grade 3 or higher adverse events was observed in the combination group, primarily characterized by neutropenia (44%). However, these events were considered manageable, and notably, no detrimental impact on quality of life was observed [77].

*TP53* genomic status does not appear to predict response to cell-cycle-targeted therapies. In many EC models, RB is expressed and appropriately controlled regardless of histologic subtype or *TP53* mutation. In *RB1* wild-type, RB-positive EC cells, preservation of RB pathway regulation, which was supported by functional testing and baseline transcriptomic profiles, has been associated with sensitivity to G1/S-directed CDK4/6 inhibitors [100]. Consistent with this, *TP53* genomic or functional status did not influence responses to agents targeting G1/S control or mitotic regulatory kinases [100]. In breast cancer, however, Kudo et al. reported that *TP53* mutations are linked to a lack of durable disease control with CDK4/6 inhibition, and that CDK2 inhibition can offset p53 loss, promoting seroconversion and disease control [101].

#### 3.1.3. Combination with CDK4/6 Inhibitor and Metformin

While the combination of hormonal therapy and CDK4/6 inhibitors has shown promising activity, therapeutic resistance remains a significant challenge. It is well-established that activation of the *PI3K* pathway promotes resistance to hormonal therapy and serves as a mechanism of adaptive response to CDK4/6 inhibition [78]. Metformin inhibits *PI3K/AKT/mTOR* signaling both directly and indirectly. Indeed, previous window-of-opportunity studies in patients with endometrial cancer have confirmed that metformin administration significantly reduces the phosphorylation of AKT and mTOR targets within the tumor [102,103]. Against this scientific background, a triplet regimen combining endocrine therapy, a CDK4/6 inhibitor, and metformin was investigated. In a phase II trial, 25 patients were treated with the combination of letrozole, abemaciclib, and metformin [78]. The study demonstrated an ORR of 32%, a 6-month PFS rate of 96.8%, and a prolonged median PFS of 19.4 months [78]. Biomarker analyses revealed that no objective responses were observed among patients with *TP53*-mutated EC or those with NSMP harboring *RB1* or *CCNE1* alterations [78]. In contrast, *CTNNB1* mutations were found to correlate with clinical benefit. All four patients identified with *ESR1* mutations achieved clinical benefit from this triplet therapy [78].

#### 3.1.4. Combination with CDK4/6i and PI3K/AKT/mTOR Pathway Inhibition

A clinical trial was conducted to evaluate the triplet combination of letrozole, abemaciclib, and the PI3K inhibitor LY3023414; however, the study was terminated prematurely due to the discontinuation of the clinical development of LY3023414 [104]. This regimen was designed to dual-inhibit the *PI3K* pathway, which is a known driver of resistance to both hormonal therapy and CDK4/6 inhibitors. At the time of termination, only five patients (four ER-positive and one ER-negative) had been enrolled. One ER-positive patient experienced a partial response to the triplet combination and remained on study treatment for over 10 months, even though she had previously progressed on letrozole plus everolimus [104]. Molecular profiling of the tumor identified an *AKT1* E17K point mutation in the pleckstrin homology domain, which is reported to promote constitutive signaling by facilitating AKT localization to the plasma membrane in a PI3K-independent manner [104]. A co-occurring *CTNNB1* mutation may also have contributed, at least in part, to the favorable response observed in this patient [104]. Alternatively, it is possible that abemaciclib helped to overcome the previous everolimus resistance, because compensatory activation of the *PI3K* and *MAPK* pathways can converge on cyclin D1 upregulation, leading to CDK4 activation, RB phosphorylation, and cell-cycle entry, a downstream program that may be effectively suppressed by CDK4/6 inhibition [104]. While the precise mechanism for overcoming resistance remains unconfirmed in this study due to the lack of baseline biopsies [104], it is noteworthy that all *CTNNB1*-mutated tumors exhibited a partial response to the doublet regimen in the related letrozole/abemaciclib study [75].

#### 3.1.5. Combination with HDAC Inhibitor

In EC cell lines, histone deacetylase (HDAC) inhibitors have been demonstrated to restore functional PR expression. Based on in vitro findings indicating that HDAC inhibitors can increase PR levels, it was hypothesized that combining these agents with hormonal therapy could prevent the ligand-induced downregulation of PR, thereby maintaining sensitivity to endocrine treatment [105]. The NRG-GY011 study was a surgical window trial involving patients with newly diagnosed endometrioid EC. In this trial, patients received either MPA monotherapy or a combination of MPA and the HDAC inhibitor entinostat for the three-week interval between diagnosis and definitive surgery. The study compared pre-treatment biopsy specimens with post-treatment surgical tissues. Although the addition of entinostat failed to demonstrate a significant inhibitory effect on PR downregulation (*p* = 0.87), a trend toward decreased Ki-67 expression was observed in the combination arm (*p* = 0.13) [105].

## 4. Future Perspectives and Expectations

### 4.1. New Agents of Endocrine Therapy

A newer class of anti-estrogen agents has been developed to overcome key resistance pathways, particularly acquired ESR1 mutations, while also addressing shortcomings of existing endocrine therapies, including tamoxifen’s partial agonist effects and the need for intramuscular fulvestrant administration [106]. The next generation of novel anti-estrogen therapies encompasses several emerging classes, including oral SERDs, proteolysis-targeting chimeras (PROTACs), complete estrogen receptor antagonists (CERANs), and selective estrogen receptor covalent antagonists (SERCAs). In the field of breast cancer, an increasing number of phase III trials have yielded positive results in both early-stage and metastatic or recurrent settings [107,108,109,110,111]. Given the hormone-dependent nature of the disease and the historical trajectory of clinical trial development, these next-generation anti-estrogen agents are highly anticipated to demonstrate efficacy in endometrial cancer as well [88]. Among these, imlunestrant (an oral SERD) is a notable example for which clinical data in endometrial cancer have already been reported [112]. In the United States, imlunestrant received approval in September 2025 for adults with advanced or metastatic ER-positive, HER2-negative breast cancer harboring *ESR1* mutations after progression on at least one prior endocrine therapy [113]. In a phase 3 EMBER-3 trial, imlunestrant demonstrated significantly longer PFS than standard therapy (exemestane or fulvestarnt) among those with ESR1 mutations but not in the overall population [110]. Moreover, Imlunestrant–abemaciclib significantly improved PFS as compared with imlunestrant, regardless of ESR1-mutation status [110]. In a phase Ia/Ib trial, imlunestrant was evaluated either as monotherapy or in combination with abemaciclib in 72 patients with ER-positive (≥1% expression) endometrioid EC who had progressed following platinum-based chemotherapy [69]. Patients with prior exposure to fulvestrant or aromatase inhibitors were excluded from this study (after 2 patients enrolled). For the imlunestrant monotherapy and combination arms, the ORRs were 10.3% and 18.2%, the CBRs were 33.3% and 42.4%, and the median PFSs were 3.8 months and 6.8 months, respectively. The safety profile demonstrated that Grade 1–2 adverse events were more frequent in the combination arm than in the monotherapy arm; the most common toxicities reported in the combination group included diarrhea (87.9%), nausea (66.7%), fatigue (48.5%), and anemia (45.5%). In exploratory analyses, no significant association was observed between *TP53* mutation status and clinical outcomes [69].

### 4.2. Ongoing Clinical Trials and Emerging Strategies (Table 3)

Clinical trials investigating endocrine therapy are also being conducted in the early-stage setting. Focusing on the favorable prognosis of the ER-positive NSMP subgroup, the randomized phase III NSMP-ORANGE trial is a treatment de-escalation study for women with stage II (with LVSI) or stage III NSMP endometrial cancer [114]. This trial compares chemoradiotherapy against radiotherapy followed by two years of progestin maintenance [114]. Additionally, the EndomERA trial (NCT05634499) is evaluating postoperative treatment with girdestrant (an oral SERD) for grade 1 endometrioid endometrial cancer.

In the advanced or recurrent setting, multiple clinical trials investigating combinations of endocrine therapy and molecularly targeted agents are currently underway (Table 3). These include studies of fulvestrant combined with PI3K inhibitors (alpelisib or copanlisib; NCT05154487, NCT05082025), MA with an AKT inhibitor (ipatasertib; NCT05538897), and the oral SERD elacestrant with or without abemaciclib (NCT07209449). Furthermore, while combinations with ICIs are anticipated to be promising, caution is necessary. In clinical trials for metastatic HR-positive breast cancer, the concurrent use of ICIs and CDK4/6 inhibitors resulted in severe and prolonged immune-related adverse events (irAEs), such as liver dysfunction and interstitial lung disease/pneumonitis [115,116]. Currently, the randomized phase II ALPINE trial (NCT06366347) is ongoing for patients with ER-positive NSMP advanced or recurrent EC. Following initial treatment with pembrolizumab, carboplatin, and paclitaxel, patients are randomized to receive maintenance therapy with either pembrolizumab monotherapy or the combination of letrozole and abemaciclib.

**Table 3:** Ongoing clinical trials regarding endocrine therapy for endometrial cancer.

Type of Class	Trial Name	Phase	Patient Population	Endocrine Therapy	Targeted Therapy
PI3K inhibitor	NCT05154487	II	ER-positive, *PIK3CA*-mutated advanced or recurrent EC	Fulvestrant	Alpelisib
	NCT05082025	II	Cohort 2: ER+ and/or PR+ *PI3K (PIK3CA, PIK3R1)* and/or *PTEN-*altered advanced or recurrent EC	Fulvestrant	Copanlisib
AKT inhibitor	NRG-GY028 NCT05538897	Ib/II	Advanced or recurrent grade 1 or 2 endometrioid EC	Megestrol Acetate	Ipatasertib
CDK4/6 inhibitor	ALPINE NCT06366347	II	ER+, MMRp, *TP53* wild (i) endometrioid endometrial cancer or (ii) endometrial carcinosarcoma with endometrioid epithelial component maintenance therapy after carboplatin + paclitaxel + pembrolizumab	Letrozole	Abemaciclib
Oral SERD	ELITE NCT07209449	II	ER+, *TP53*wt, No known MMRd or *POLE* mutation advanced or recurrent EC	Elacestrant	+/− Abemaciclib
	EndomERA NCT05634499	II	Grade 1 endometrioid EC	Girdestrant	-
Progestin	NSMP-ORANGE NCT05255653-3	III	ER+ Stage II (with LVSI) or Stage III NSMP EC	Progestin	-

Abbreviations: EC, endometrial carcinoma; ER, estrogen receptor; PR, progesterone receptor; MMRp, mismatch repair proficient; wt, wild-type; LVSI, lymphovascular space invasion; NSMP, no specific molecular profile; SERD, selective estrogen receptor degrader; MMRd, mismatch repair deficiency.

## 5. Challenges

### 5.1. Who Is the Appropriate Candidate for Endocrine Based Therapy?

Careful patient selection is essential to maximize the benefit of endocrine therapy and likely requires consideration of both histopathologic and genomic characteristics [88]. Nevertheless, major issues persist, including endocrine therapy resistance and an incomplete understanding of the factors governing sensitivity and resistance [88]. In this context, relying on eligibility criteria defined only by ER or PR overexpression may not provide the most reliable prediction of response [88]. Lack of predictive biomarkers beyond ER/PR immunohistochemistry is problematic.

### 5.2. Challenges in ER Assessment

Challenges in ER assessment for pathological diagnosis remain. Although ER evaluation is relatively straightforward, optimal clinical application requires awareness of specific limitations. Historically, a 5% cutoff has been used in some earlier studies [117,118]; however, the most recent guidelines and clinical trial consensus recommend a threshold of ≥10% [15,42,117]. In recent years, studies have also been conducted to determine thresholds using the Allred score, a scoring system combining staining intensity and percentage of positive cells that is continuously used in breast cancer [29,41]. Further validation of the optimal cutoff will require dedicated, rigorously standardized IHC assessment methodologies in the context of EC, including whole-slide evaluation. While molecular classification can be reliably performed on diagnostic endometrial biopsy or curettage specimens [119,120], ER expression may differ between primary and metastatic sites [121,122,123]. Therefore, in advanced and recurrent EC, re-biopsy of a metastatic lesion and repeat ER/PR testing is recommended when feasible before enrollment in endocrine therapy trials [123,124]. Prior reports have indicated that ER and PR expression by IHC tends to be reduced in metastatic lesions compared with primary tumors [121,125]. In endometrioid EC, ER-IHC levels in distant metastases were reported to be significantly lower than those in intra-abdominal metastatic sites [121,125].

In the PROMOTE study, an observational study of 80 patients with EC who had received at least one prior line of endocrine therapy, an analysis of pre-treatment tumor biopsies revealed that lymph node metastases tended to show lower PR-IHC scores and ER pathway activity scores (ERPASs) compared with other metastatic locations [126].

### 5.3. Predictive Biomarkers for Precision Patient Selection

From the perspective of precision medicine, the combination of endocrine therapy and CDK4/6 inhibitors may be recommended for patients with *TP53* wild-type (NSMP), ER-positive endometrioid EC. Regarding molecular subtypes, the highest clinical efficacy is observed in the “CN-L or “NSMP” groups [75,76]. In contrast, patients in the “CN-H” or serous-like groups, which typically harbor *TP53* mutations, derive minimal clinical benefit from these regimens [75,76]. Furthermore, the presence of *CTNNB1, KRAS,* or *CDKN2A* mutations may identify tumors with a high dependency on the CDK4/6 pathway, potentially serving as predictive biomarkers for favorable responses to this combination therapy [75,76].

### 5.4. When Is the Appropriate Timing of Hormonal Therapy?

Although a randomized crossover trial intended to compare endocrine therapy and chemotherapy was prematurely terminated due to insufficient patient accrual [83], a confirmatory phase III trial remains essential to definitively establish the therapeutic position of hormonal therapy [77]. Considering the significantly more favorable clinical outcomes observed in chemotherapy-naïve patients compared to those who have received prior treatment [72], evaluating endocrine-based strategies in the first-line setting is increasingly warranted.

## 6. Conclusions

While endocrine therapy has long been a staple in the management of endometrial cancer, the emergence of novel molecular classifications and the expanding repertoire of hormonal and targeted agents, coupled with the accumulation of promising clinical trial data, now necessitate a definitive solidification of its role within the modern treatment landscape. The integration of TCGA-based molecular profiling has enabled the identification of the NSMP subgroup as the primary population eligible for endocrine-based strategies. Current evidence suggests that combining ET with CDK4/6 or mTOR inhibitors provides superior clinical benefit compared to traditional monotherapy, particularly in the chemotherapy-naïve setting where exceptionally prolonged responses have been observed. Moving forward, the primary challenge lies in refining patient selection through robust predictive biomarkers beyond ER/PR immunohistochemistry, such as *CTNNB1, RB1,* and *CCNE1* status. Confirmatory phase III trials are essential to transition these combinations from “investigational” to “standard-of-care” status. Furthermore, the clinical application of next-generation agents like oral SERDs and the exploration of optimal sequencing with immune checkpoint inhibitors will be pivotal in improving long-term outcomes and quality of life for patients with advanced EC. Ultimately, endocrine therapy is transitioning from a low-intensity alternative into a cornerstone of precision oncology in EC.

## Figures and Tables

**Table 1:** Estrogen receptor expression in endometrial cancer by each histological type and molecular subtype.

Histology	ER Positivity (%)	References
Endometrioid carcinoma, grade 1	83.8–89.7	[43,44]
Endometrioid carcinoma, grade 2	73.5–81.5	[43,44]
Endometrioid carcinoma, grade 3	25.0–78.2	[43,44,45,46]
Serous carcinoma	21.2–64.6	[45,47,48]
Clear cell carcinoma	0–12.8	[49,50]
Dedifferentiated/Undifferentiated carcinoma	31.0	[51]
Carcinosarcoma	8.0	[52]
Mesonephric-like adenocarcinoma	24.0	[53]
Gastric-type adenocarcinoma	0	[54]
**Molecular subtype**	**ER positivity (%)**	**References**
*POLE*mut	66.7–75.1	[42,55]
dMMR	64.8–90.0	[42,55,56,57,58]
NSMP	80.6–95.9	[29,41,42,55,56,57,58]
p53abn	50.3–68.5	[42,55,56,58]

Abbreviations: dMMR, mismatch repair deficiency; NSMP, non-specific molecular profile. Note: For each subtype, data from studies with 10 or more cases were referenced.

## Data Availability

No new data were generated or analyzed in this study. All data cited are available in the original publications referenced in this article.
